# 

**Published:** 1899-09

**Authors:** 


					﻿Vol. LIII.	SEPTEMBER, 1899.	No. 9.
THE
DENTAL REGISTER
A MONTHLY JOURNAL.
Devoted to the Interests of the Profession.
EDITED BY
J. TAFT, D.D.S.
PUBLISHED BY
SAM’L A. CROCKER & CO.,
OHIO DENTAL AND SURGICAL DEPOT,
35, 37 and 39 West Fifth Street,	CINCINNATI, OHIO.
Entered at the Postoffice at Cincinnati, O., as second class mail matter.
TERMS: $2.00 per Annum in Advance. Single Copies, 25 Cts.
				

